# Homologue Pairing in Flies and Mammals: Gene Regulation When Two Are Involved

**DOI:** 10.1155/2012/430587

**Published:** 2011-12-22

**Authors:** Manasi S. Apte, Victoria H. Meller

**Affiliations:** Department of Biological Sciences, Wayne State University, Detroit, MI 48202, USA

## Abstract

Chromosome pairing is usually discussed in the context of meiosis. Association of homologues in germ cells enables chromosome segregation and is necessary for fertility. A few organisms, such as flies, also pair their entire genomes in somatic cells. Most others, including mammals, display little homologue pairing outside of the germline. Experimental evidence from both flies and mammals suggests that communication between homologues contributes to normal genome regulation. This paper will contrast the role of pairing in transmitting information between homologues in flies and mammals. In mammals, somatic homologue pairing is tightly regulated, occurring at specific loci and in a developmentally regulated fashion. Inappropriate pairing, or loss of normal pairing, is associated with gene misregulation in some disease states. While homologue pairing in flies is capable of influencing gene expression, the significance of this for normal expression remains unknown. The sex chromosomes pose a particularly interesting situation, as females are able to pair X chromosomes, but males cannot. The contribution of homologue pairing to the biology of the X chromosome will also be discussed.

## 1. Introduction

One of the most intriguing aspects of somatic homologue pairing is that such a basic condition has enormous variability between species. Homologues pair vigorously in *Drosophila*, as illustrated by the remarkable alignment of polytene chromosomes. In fact, homologue pairing is pervasive throughout the Diptera, but in other organisms the occurrence and extent of homologue pairing is often unknown [1, 2]. Close association of homologous chromosomes in vegetative diploid budding yeast has been reported, but a careful reexamination suggested that little, if any, pairing occurs [3]. In diploid fission yeast both homologues occupy the same chromosome territory and centromeric pairing is observed in most cells [4]. Early studies suggested somatic homologue pairing in numerous plant species (Reviewed in [2]). Recent work supports the idea of homologue pairing in some grains and fungi, but also casts doubt on other reports of pairing in plants [5–8].

## 2. Mammals: Pairing to Share Information

**Figure 1:**
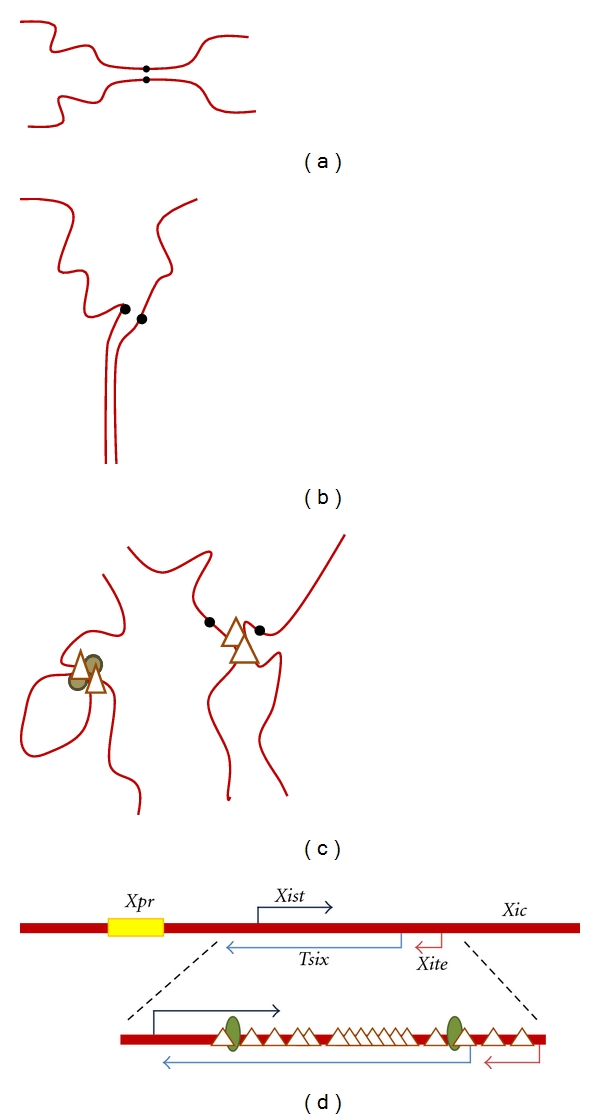
Modes of somatic pairing in mammalian tissues. (a) Pericentromeric homologue pairing in parts of the brain. Centromeres are depicted by black dots. (b) Abnormal pairing of chromosome 19q in renal carcinoma. (c) Looping between two sites on a chromosome (left) and interchromosomal contacts (right) are mediated by sequence-specific DNA-binding proteins such as CTCF (triangle) and cohesin (brown circle). (d) Pairing of the *X inactivation center* (*Xic*) initiates X chromosome inactivation in females. Sequences that participate in *Xic *pairing are depicted. The *X-pairing region *(*Xpr*, yellow) initiates *Xic* pairing. *Tsix* (light blue) and *Xite *(pink) pair transiently, enabling counting and choice to occur. Oct4 and CTCF are necessary for contact and communication at the *Xic*. Oct4-binding sites (green ovals) and CTCF-binding sites (triangles) within the *Tsix *and *Xite* regions of the mouse *Xic* are depicted.

Mammals have perhaps the most elaborate manifestation of homologue pairing. While complete pairing of the mammalian genome is not reported outside of the germline, somatic pairing of specific chromosomal regions does occur, but is tightly regulated. For example, homologous association of pericentromeric regions of human chromosome 1 is detected in cerebellar, but not cerebral, tissue [9]. Heterochromatic regions of chromosomes 8 and 17 also pair in parts of the brain (Figure 1(a)) [10, 11]. Chromosome-specific pairing of chromosome 7 and 10 is also seen in case of cell line derived from follicular lymphoma [12]. Several cell lines derived from renal carcinomas display an abnormal pairing of one arm of chromosome 19 and misexpress genes within the paired region (Figure 1(b)) [13]. This suggests that modulation of homologue associations may be necessary for normal gene regulation. The mechanism of pairing in these examples has not been investigated. However, this type of pairing is very tissue specific and limited to portions of particular chromosomes. It therefore must depend on chromosome-specific features, as well as developmental cues.

The best understood somatic homologue associations in mammalian cells are transient and occur at individual loci, rather than encompassing extensive chromosomal regions. These contacts appear to be a subset of long-range interactions between chromosomes, which includes looping and interactions between nonhomologous regions (Figure 1(c)) [14, 15]. One notable function of these interactions is their role in establishing inactivation of one of the two female X chromosomes and in controlling monoallelic expression of imprinted genes.

The long-range contacts made by mammalian homologues overlay a general nuclear organization that seems designed to discourage interaction. Mammalian chromosomes occupy nonoverlapping regions, termed chromosome territories, in the nucleus. These territories are organized by specific rules (Reviewed by Spector [16]). For example, gene-poor regions tend to be close to the nuclear membrane, while gene-dense chromosomes localize in interior of the nucleus [14, 17]. The territories of small and early replicating chromosomes also tend to be interior. Interestingly, in human epithelial cancer cell lines and mouse primary lymphocytes the territories occupied by the homologues are more widely separated than expected from a random distribution [18, 19]. One function of chromosome territories may be to keep the homologues apart.

The properties of the molecules that mediate long-range contacts between allelic and nonallelic loci suggest strategies that facilitate specific interactions. One of these molecules is CTCF (CCCTC-binding factor), a highly conserved, DNA-binding protein with a multitude of seemingly disparate regulatory functions (Reviewed by Philips and Corces [20]). Depending on context and binding partners, CTCF can be a transcriptional repressor or an activator [21–24]. Adjacent CTCF binding sites are often drawn into chromatin loops, insulating promoters from nearby regulatory regions [25–30]. One of the best-understood examples is found at the imprinted Igf2/H19 locus. Imprinting, established in the parental germline, produces an allele-specific difference in genetic properties (Reviewed by Verona et al. [31]). The Igf2/H19 locus has a CTCF-binding site that is differentially methylated in the parental germlines [32–34]. Methylation of the paternal allele blocks CTCF binding, preventing formation of an insulator that would otherwise separate Igf2 from an enhancer [33, 35–37]. On the maternal allele, CTCF binds between Igf2 and this enhancer, silencing Igf2 by insulation and through recruitment SUZ12, a member of the Polycomb Repressive Complex 2 (PRC2) [29]. On the maternal chromosome CTCF binding adjacent to H19 is necessary to induce expression of this transcript [38].

CTCF also mediates interactions between Igf2/H19, on chromosome 7, and other regions throughout the genome. Igf2/H19 contacts the Wsb1/Nf1 locus on chromosome 11 [26, 39]. This interaction is dependent upon binding of CTCF to the maternal Igf2/H19 allele and is required for monoallelic expression from Wsb1/Nf1. Additional interactions between Igf2/H19 and several other imprinted loci have been identified, and these findings are consistent with the idea that Igf2/H19 coordinates the epigenetic status of imprinted regions throughout the genome [40].

Some imprinted homologues pair transiently, an activity that may be necessary for normal developmental regulation. In lymphocytes, transient association at 15q11–q13 occurs in late S phase [41]. This region is imprinted, containing several monoallelically expressed genes. Loss of expression, or lack of normal imprinting at this locus, causes Prader-Willi and Angelman syndromes, both of which display developmental and neurological abnormalities (Reviewd by Lalande [42]). Interestingly, lymphocytes from Prader-Willi and Angelman syndrome patients do not pair [41]. Homologue communication at 15q11-q13 may be a factor in normal brain development, as this locus pairs persistently in normal brain, but not in brains from patients with some autism-spectrum disorders [43].

Homologue pairing also plays a central role in orchestration of X inactivation in mammalian females. Mammalian females randomly inactivate one X chromosome, thus maintaining an equivalent ratio of X to autosomal gene products in both sexes [44, 45]. Each cell of the early embryo counts the number of X chromosomes and inactivates all but one (Reviewed by Royce-Tolland and Panning [46]). Counting, and choice of the inactive X, relies on a transient pairing of the *X inactivation center* (*Xic*), a locus on the X chromosome (Figure 1(d)). Pairing is believed to enable XX cells to coordinate inactivation of a single X chromosome. Deletion of regions engaged in pairing led to skewed or chaotic X inactivation [47]. The process of pairing is complex, involving multiple elements within the *Xic*. The *X-pairing region* (*Xpr*) may support initial interactions, and its deletion diminishes *Xic* pairing [48, 49]. Several genes within the *Xic* produce noncoding RNAs that participate in counting and inactivation of the X chromosome. *Xist*, a long noncoding RNA, initiates the process of X inactivation and coats the inactive X (Reviewed by Chow and Heard [50]). *Tsix*, transcribed antisense to *Xist*, and a nearby gene *Xite* contribute to pairing of the *Xic* and also produce noncoding RNAs (Reviewed by Lee [51]). Following pairing, transcription of *Tsix *and *Xite* is necessary for orderly X inactivation, suggesting that communication might occur by an RNA-protein bridge between two X chromosomes [52]. CTCF plays a central role in pairing at the *Xic*. The *Tsix* promoter contains numerous CTCF binding sites (Figure 1(d)) [52–55]. Pairing at the *Xic* is disrupted upon the loss of CTCF [56]. Initiation of inactivation occurs during a narrow window in early development [57]. Oct4, a transcription factor key to the maintenance of stem cells, forms a complex with CTCF at *Tsix, *and is required for transient association of *Xics* [56]. After this transient pairing, the X chromosomes separate, assume different fates and localize to distinct nuclear compartments.

The examples above illustrate the idea that CTCF fulfills disparate functions in a developmental and cell type-specific manner. The proteins mentioned above, Oct4 and SUZ12, are among many CTCF partners that enable modulation of CTCF effects [58]. An additional CTCF binding protein that contributes to its localization and function is nucleophosmin, a component of the nucleolus [59]. Some loci that bind CTCF are anchored at the nucleolus, leading to the idea that the nucleolus functions as a hub where long-range interactions occur. While recruitment to the nucleolus appears to be a factor for some CTCF-bound loci, it does not contribute to X chromosome pairing [59, 60].

Another protein that contributes to CTCF function is cohesin, a multisubunit complex that regulates sister chromatid cohesion during meiosis and mitosis. Cohesin, consisting of SMC1, SMC3, Scc1, and Scc3 subunits, is believed to encircle sister chromatids to maintain their association [61, 62]. The C-terminus of CTCF interacts with the cohesin subunit Scc3, and cohesin and CTCF are often colocalized on mammalian chromosomes [63–65]. Depletion of CTCF results in loss of cohesin binding but, at most sites, loss of cohesin does not affect CTCF binding to DNA [66, 67]. CTCF thus appears to recruit cohesin to specific DNA sequences. Cohesin recruitment facilitates long-range interactions, either by securing aligned regions or by inducing looping. For example, cohesin plays a regulatory role in CTCF-mediated intrachromosomal contacts between sites in the interferon-*γ* locus [65, 66]. Loss of cohesin or CTCF also leads to misregulation of expression from Igf2/H19 [39, 64].

While cohesin colocalizes with CTCF on mammalian chromosomes, the association of these molecules is not universal. In *Drosophila*, cohesin and CTCF have not yet been shown to colocalize. In spite of this, in flies CTCF performs many functions similar to those in mammals. For example, it localizes to insulators and contributes to looping between boundary elements [68, 69]. *Drosophila* CTCF also plays a role in imprinting in flies [70].

## 3. Flies: Always in Touch

In contrast to the carefully orchestrated pairing of specific loci in mammals, complete homologue pairing is the default condition in *Drosophila*. Pairing is evident from the mitotic cycle 13 of embryogenesis onwards [71, 72]. Cellularization occurs during cycle 14, which marks a dramatic reorganization of the nucleus [73]. Heterochromatin becomes detectable at cycle 14, and transcription of zygotic genes begins in earnest [74]. While pairing is persistent throughout the cell cycle from this point onwards, it is relaxed, but still apparent, during replication and mitosis [75, 76].

Homologues might encounter each other by directed movement, or by random diffusion [77]. Analysis of chromosomal movements preceding pairing in embryos supports the idea that random motion leads to homologue encounters and suggests independent initiation at numerous sites, rather than a processive zippering along the length of the chromosome [71, 75]. Space constraints within a chromosome territory or an underlying chromosome arrangement could speed the search. Early studies by Rabl and Boveri revealed the nonrandom organization of the interphase nucleus. The centromeres cluster at one pole of the nucleus, while the chromosome arms extend across the nucleus towards the other pole. This polarized pattern of chromosomal arrangement, known as Rabl configuration, is not apparent in some species (rice, maize, mouse, and humans) but is observed in a wide range of organisms (*S. cerevisiae*, *S. Pombe, Drosophila*, and several grains) (Reviewed by Spector [16] and Santos and Shaw [78]). The Rabl configuration is reminiscent of the arrangement of chromosomes following mitosis, where the centromeres lead the chromosomes into the daughter cells. While the anaphase movement of chromosomes does promote this arrangement, cell division is not essential for the Rabl conformation in yeast [79]. Regardless of how formed, homologous chromosomes in the Rabl configuration are roughly aligned, more or less parallel, placing alleles closer together than predicted by chance distribution.

While pairing of imprinted loci and the *Xic* is necessary for correct regulation of developmentally important genes in mammals, there are no examples of flies utilizing chromosome pairing to count X chromosomes or to regulate monoallelic gene expression. However, homologue pairing in flies does affect gene expression through a mechanism known as transvection [80]. Pioneering work by Lewis on the *Ultrabithorax* (*Ubx*) gene showed that the mutant phenotype was stronger when pairing between two loss-of-function *Ubx* alleles was disrupted by chromosomal re-arrangements. When paired, *Ubx* expression was elevated, enabling complementation between the two mutations. A well-supported model for transvection is that pairing enables regulatory elements on one chromosome to drive (or silence) expression from an intact promoter on the other chromosome [81]. Confirmation of transvection is obtained when the phenotype is sensitive to disruption of pairing, for example, by inversion of one chromosome [80, 82]. Transvection has been demonstrated for numerous genes in *Drosophila*, and it appears able to operate throughout the genome [83]. Transvection has also been observed in the diploid stages of *Neurospora* [5]. A few examples of transvection have been described in mammals, and the term is often used to describe nonallelic regulatory interactions *in trans*, such as the CTCF-mediated long-range interactions that were described in preceding sections [84, 85].

A limitation of our understanding of transvection is how alleles communicate. Communication may differ from gene to gene. For example, transvection at *Ubx* is disrupted by breaks anywhere within a large critical region between* Ubx* and the centromere, but transvection at the *yellow* gene is only sensitive to breaks very close to the gene. This is consistent with different mechanisms of pairing or communication at these loci, but could also reflect the length of the cell cycle, and thus the time available for homologue association, at the time of gene expression [86]. For example, expression of *Ubx* is required in rapidly cycling embryonic cells. In contrast, the critical period for *yellow* expression is in pupal cells that have ceased dividing. In accordance with this idea, extension of the cell cycle in *Ubx* mutants with inversions reduces phenotypic severity, presumably by allowing extended time for chromosome pairing [86].

One molecule that affects pairing-dependent gene regulation is encoded by *zeste* (*z*). Zeste is a DNA-binding protein that affects pairing-dependent expression at many genes that display transvection (Reviewed by Pirrotta [87] and Duncan [88]). The Zeste protein polymerizes, leading to the suggestion that it might bridge homologues, but loss of Zeste does not affect homologue pairing [89]. Zeste binding sites are found in promoters, and the Zeste protein interacts with the activating *Trithorax *chromatin regulatory complex, as well as the repressing *Polycomb* PRC1 complex [90, 91]. Thus it appears likely that Zeste is a transcription factor able to interpret the state of homologue pairing.

An RNAi screen in tissue culture cells identified Topoisomerase II (Top2) as necessary player in homologue pairing [76]. Topoisomerases play pivotal roles by solving topological problems associated with DNA replication, transcription, recombination, repair, and chromosome segregation (Reviewed by Nitiss [92]). Type II topoisomerases introduce double-strand breaks, pass an intact DNA duplex through the cut, and rejoin the cut ends. Top2 also makes up a large fraction of the insoluble nuclear matrix and contributes to chromosome architecture [93, 94]. It preferentially binds scaffold-associated regions, which anchor chromatin loops during interphase. There are several potential mechanisms through which Top2 might contribute to pairing. Because it plays a central role in chromosome organization, loss of Top2 could lead to a general disruption that abrogates homologue association. It is also possible that Top2 engages in protein/protein interactions that stabilize pairing.

One protein that interacts with Top2 and also affects pairing in *Drosophila*, is condensin. Condensins function in chromosome condensation, induction of DNA supercoiling, and anaphase chromosome segregation. Metazoans have two paralogous condensin complexes, condensin I and II. Each contains conserved SMC2 and SMC4 subunits, but different non-SMC subunits: Cap-H, Cap-G, and Cap-D2 or Cap-H2, Cap-G2, and Cap-D3 [95, 96]. Condensins influence the activity of Top2, and Top2 interacts directly with the *Drosophila* Cap-H homologue Barren on mitotic chromosomes [97]. Both proteins are necessary for chromosome segregation, and loss of either produces a similar mitotic defect. Condensin I is also required for localization of Top2 on mitotic chromosomes in flies, yeast, and humans [98–100].

In spite of the dependent interactions between condensin and Top2, condensin acts to antagonize homologue pairing in *Drosophila* [101]. Most dramatically, ectopic expression of Cap-H2 in salivary glands separates the aligned polytene chromosomes. Increased condensin reduces transvection at two loci, revealing the dissociation of paired homologues in diploid cells. The involvement of Top2 and condensin reveals that homologue pairing in flies is regulated by conserved proteins necessary for the maintenance of chromosomal architecture and stability in all eukaryotic organisms. It will be fascinating to see if Top2 or condensin levels affect pairing in other organisms.

## 4. Pairing and Sex Chromosomes

An unanswered question is whether pairing-dependent regulation contributes to the expression of wild-type genes in *Drosophila*. Analysis of *Ubx* revealed that expression from a wild-type allele was increased when it could pair with a gain of function mutation [102]. Homologue pairing might also contribute to expression of other unmutated genes in a wild-type context. The phenotypic normality of flies with inverted chromosomes would suggest that transvection makes little contribution to expression, but a functional assay for homologue association demonstrated that alleles on inverted chromosomes can pair surprisingly efficiently, when given sufficient time [86]. But there are situations in which homologue pairing cannot occur, including the single male X chromosome and regions made hemizygous by deficiency. If pairing influences expression of wild type genes, the regulation of the entire X chromosome might differ between the sexes. This could contribute to sexually dimorphic expression or influence the biology of the X chromosome.

Flies have a dedicated regulatory system that accommodates hemizygosity of the X chromosome in males. Males produce the chromatin-modifying Male-Specific Lethal (MSL) complex, which is recruited to the X chromosome at 3 h after fertilization [103]. The result is increased expression of virtually every X-linked gene. Surprisingly, RNA sequencing of single-sexed embryos has identified partial dosage compensation at mitotic cycle 13, an hour before the MSL complex localizes to the X chromosome [104]. One mechanism proposed to explain this is that pairing of X chromatin in females inhibits transcription from X-linked genes. This idea deserves to be tested, as it could explain several situations in which dosage compensation occurs in the absence of the MSL complex. For example, X-linked genes are dosage compensated in the male germline, where the MSL complex is not formed [44, 105]. Autosomal deficiencies are partially compensated by an unknown mechanism [106]. In addition, considerable evidence supports the idea that the MSL complex does not fully compensate X-linked genes in somatic cells. If formation of the MSL complex is blocked, expression of X-linked genes is reduced by 25%–30%, rather than the predicted 50% [107, 108]. These observations support the idea that differences in gene copy number are buffered by mechanisms that operate throughout the genome (Reviewed by Stenberg and Larsson [106]).

**Figure 2:**
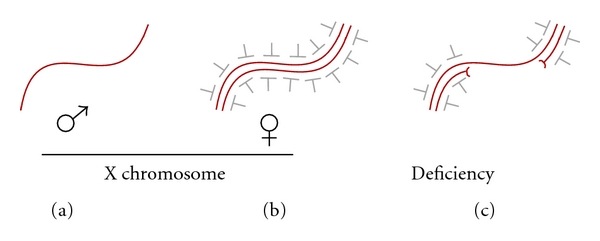
Hypothetical model for pairing-dependent buffering of gene dosage in flies. (a) The unpaired X chromosome of males escapes repression. (b) Paired female X chromosomes are subject to repression. (c) Paired regions of an autosome are repressed, but an unpaired region created by deficiency escapes repression.

A copy number buffering mechanism would differentially affect X-linked gene expression in males and females. Over time, this could be a factor in creation of the striking differences in gene distribution observed when comparing the X chromosome and the autosomes in some species (Reviewed by Vicoso and Charlesworth [109] and Gurbich and Bachtrog [110]). For example, the mammalian X chromosome appears enriched for genes with a male-biased expression, including those expressed in the premeiotic testes [111]. This is postulated to reflect the fact that hemizygosity of the male X chromosome enables rapid selection for beneficial recessive alleles. The same argument should apply to other species with XY males, including flies. However, the X chromosomes of *Drosophila melanogaster* and related species are depleted for genes with male-biased expression in somatic tissues and testes and enriched for genes with female-biased expression [112]. These notable differences in the distributions of sex-biased genes in mammals and flies have yet to be adequately explained. A recent study revealed that the fly X chromosome was also depleted for developmentally regulated genes, with the notable exception of those expressed in the ovary [113]. The authors propose that demasculinization of the X chromosome was due in part to the fact that male-biased genes tend to be developmentally regulated and suggest that chromatin modification by the MSL complex may be incompatible with developmental regulation, making the X chromosome an unfavorable environment. However, a genome-wide buffering system that contributes to X chromosome dosage compensation could also influence the distribution of developmentally regulated genes. Analysis of expression in flies with autosomal deficiencies and duplications lends support to the idea that such a system exists, but constitutively expressed genes and those with highly regulated expression respond differently [114]. A speculative model for the role of homologue pairing in buffering gene dose is presented in Figure 2. A key feature of our model is that homologue pairing is repressive. The absence of pairing of the male X chromosome, and autosomal deficiencies, leads to a modest increase in expression from these regions.

## 5. Conclusions

Somatic chromosome pairing obeys strikingly different rules in mammals and flies. Mammals sharply limit contacts between homologues. When homologues do make contact it often serves to coordinate regulatory mechanisms, such as imprinting and X inactivation, that are essential for normal development. It seems ironic that mammals use pairing to communicate critical information, yet flies, with constant homologue pairing, appear to make little use of this feature of genome organization. Recent studies of early dosage compensation and buffering of copy number variation in flies suggest that additional regulatory mechanisms exist to accommodate variation in gene dosage. A pairing-based regulation of gene expression could account for many of the findings of these studies. A broader question is why homologue pairing exists in some species, but not in others. The precise control of homologue association in mammals, and inappropriate pairing in some cancers, suggests that homologue association can be dangerous. What this danger is, and how flies evade it, remains to be discovered.
